# Whole genome sequence data of *Mycobacterium tuberculosis* XDR strain, isolated from patient in Kazakhstan

**DOI:** 10.1016/j.dib.2020.106416

**Published:** 2020-10-17

**Authors:** Asset Daniyarov, Askhat Molkenov, Saule Rakhimova, Ainur Akhmetova, Zhannur Nurkina, Dauren Yerezhepov, Lyailya Chingissova, Venera Bismilda, Bekzat Toxanbaeva, Ainur Akilzhanova, Ulan Kozhamkulov, Ulykbek Kairov

**Affiliations:** a Laboratory of Bioinformatics and Systems Biology, Center for Life Sciences, National Laboratory Astana, Nazarbayev University, Nur-Sultan 010000, Kazakhstan; b RSE National Center for Biotechnology, Nur-Sultan 010000, Kazakhstan; c Laboratory of Genomic and Personalized Medicine, Center for Life Sciences, National Laboratory Astana, Nazarbayev University, Nur-Sultan 010000, Kazakhstan; d L.N.Gumilev Eurasian National University, Nur-Sultan 010000, Kazakhstan; e National Scientific Center of Phthisiopulmonology of the Republic of Kazakhstan, Almaty 050000, Kazakhstan

**Keywords:** Mycobacterium, Whole genome sequence, Draft genome, Extensively drug-resistance, Tuberculosis, Kazakhstan

## Abstract

Drug-resistant tuberculosis (TB) is a major public health problem. Clinical *Mycobacterium tuberculosis* (MTB) isolate with Extensively drug-resistant tuberculosis (MTB-XDR) profile was subjected to whole-genome sequencing using a next-generation sequencing platform (NGS) Roche 454 GS FLX+ followed by bioinformatics sequence analysis. Quality of read was checked by FastQC, paired-end reads were trimmed using Trimmomatic. *De novo* genome assembly was conducted using Velvet v.1.2.10. The assembled genome of XDR-TB-1599 strain was functionally annotated using the PATRIC platform. Analysis of *de novo* assembled genome was performed using ResFinder, CARD, CASTB and TB-Profiler tools. MIRU_VNTR genotyping on 12 loci and spoligotyping have been performed for XDR-TB-1599 isolate. *M. tuberculosis* XDR-TB-1599 strain yielded an average read depth of 21-fold with overall 4 199 325 bp. The assembled genome contains 5528 protein-coding genes, including key drug resistance and virulence-associated genes and GC content of 65.4%. We identified that all proteins encoded by this strain contain conserved domains associated with the first-line anti-tuberculosis drugs such as rifampicin, isoniazid, streptomycin and ethionamide. TB-Profiler had higher average concordance results with phenotypic DST (drug susceptibility testing) in comparison with ResFinder, CARD, CASTB profiling to first-line (75% vs 50%) and second-line (25% vs 0%) of anti-TB drugs, correspondingly. To our knowledge, this is the first report of a highly annotated and characterized whole-genome sequence and *de novo* assembled XDR-TB *M.tuberculosis* strain isolated from a sputum of new TB case-patient from Kazakhstan performed on Roche 454 GS FLX+ platform. This report highlights an important role of whole-genome sequencing technology and analysis as an advanced approach for drug-resistance investigations of circulated TB isolates.

**Specifications Table**

**Table:** 

Subject	Genetics, Genomics and Molecular Biology
Specific subject area	Microbiology, genomics and bioinformatics
Type of data	Whole-genome sequence data in FASTA format, figure, tables.
How data were acquired	Genome sequencing with next-generation sequencing platform Roche 454 GS FLX+ at National Laboratory Astana.
Data format	Raw sequencing reads (fastq), analyzed and assembled genome sequence (fasta)
Parameters for data collection	Whole-genome sequencing was performed on genomic DNA from a sputum
Description of data collection	*M. tuberculosis* XDR-TB-1599 was isolated from sputum samples of clinical patients with pulmonary tuberculosis in Kazakhstan. DNA was extracted from *M.tuberculosis* cultures by the cetyl-trimethyl ammonium bromide (CTAB) method. The DNA quality and quantity were assessed by Qubit 3.0 Fluorometer with dsDNA Broad Range Assay kit (Thermo Fisher Scientific) and agarose gel electrophoresis. DNA libraries were prepared by using a GS FLX Titanium rapid library preparation kit. A prepared genomic library of TB strain was sequenced on Roche 454 GS FLX+ Titanium NGS platform at the Center for Life Sciences, Nazarbayev University, Kazakhstan. The genome was assembled using Velvet, variant calling by GATK tools, annotated with PATRIC.
Data source location	Center for Life Sciences, Nazarbayev University, Nur-Sultan (Astana), Kazakhstan / National scientific center of phtysiopulmonology, Almaty, Kazakhstan Nur-Sultan, Almaty Kazakhstan Latitude and longitude (and GPS coordinates): 51.092115, 71.396840 & 51° 5′ 31.614′' N, 71° 23′ 48.624′' E.
Data accessibility	Repository name: Sequence Read Archive Data identification number: PRJNA481625 BioSample accession: SAMN09685213 Data is publicly available at NCBI Genbank from the following links: https://www.ncbi.nlm.nih.gov/bioproject/PRJNA481625 https://www.ncbi.nlm.nih.gov/biosample/9685213 The raw data in fasta and fastq formats represented by genomic DNA sequences of *M. tuberculosis* XDR-TB-1599 isolate (raw_data_xdr-tb-1599) and the dataset represented by genomic DNA sequences of reference strains of *M. Tuberculosis* (XDR-TB-1599_with_other_MTB-isolates) are available at https://github.com/LabBandSB/mtb-xdr-dataset

## Value of the Data

• The dataset provides information on genomic variants in whole-genome sequence of extensively drug-resistant *M. tuberculosis* XDR-TB-1599 strain.
• Whole-genome sequence data from extensively drug-resistant strain could be useful for comparative genomic analysis of *M. tuberculosis* strains with other types of drug-resistant (susceptible and resistant) isolated in different countries.
• *M. tuberculosis* XDR-TB-1599 strain has been genotyped by MIRU-VNTR and spoligotyping approaches and provides complementary information for further investigations by researchers.
• Raw whole-genome sequencing data available for the biomedical community and can be pre-processed by different bioinformatics pipelines and analysed with whole-genome data of other species for evolutionary studies of genetic variability in drug-resistant genes.

## 1 Data Description

Tuberculosis (TB) especially multidrug-resistant TB (MDR-TB) and extensively drug-resistant TB (XDR-TB) caused by *M.tuberculosis* bacteria continues to be a public health problem in many countries. Despite progress in decreasing the global incidence of tuberculosis, the existence of multidrug-resistant (MDR-TB) and extensively drug-resistant (XDR-TB) tuberculosis has led to an increase in the number of MDR cases in some countries, including Kazakhstan. Kazakhstan within the top 20 countries with high MDR-TB burden according to WHO Report 2019 [1]. It is extremely important to examine susceptible and resistant strains with different mutations in genes encoding drug metabolism among *M.tuberculosis* isolates. Previous investigations of *M.tuberculosis* isolates circulated in Kazakhstan have been performed using Sanger sequencing, spoligotyping, MIRU-VNTR typing and NGS approaches [2], [3], [4]. Here, we report the whole genome sequence and analysis of XDR-TB strain, performed by next-generation sequencing platform. The raw dataset was deposited in the NCBI Sequence Read Archive under PRJNA481625.

The draft genome of XDR-TB-1599 contains 1160 contigs with an estimated assembled genome size of 4 199 325 bp, 5528 coding sequences (CDS), 65.41% GC content, 43 rRNAs and 4 tRNAs (Table 1).

**Table 1:** Isolate characteristics: data derived from WGS including mapping indicators.

Isolate	SNPs														INDELs^a^			Mapping Indicator^b^
	Non-synonymous mutations (Ns)				S	T_c_		IM		T		N_s_/S Ratio		T_c_/ T	T		Size Range	Coverage (%)
	T	PPE	PE	All														
XDR-TB-1599	599	53	36	510	359		921		37		958		1.6685	0.9614	183	1–364		94.43

a Small INDELs identified using GATK 4.1.4.1;.

b Relative to *M. tuberculosis* H37Rv; T - total number of variants; PE - In PE family genes; PPE - In PPE family genes; All - In all other; IM - Intergenic mutations; PE genes, genes with the N-terminal proline-glutamine motif; PPE genes, genes with the N-terminal proline-glutamine-glutamine motif; N_s_/S Ratio, the ratio of nonsynonymous (N_S_) and synonymous (S) variants; T_c_/ Total, the ratio of SNPs in coding regions (T_c_) and the total number of SNPs.

**Fig. 1:**
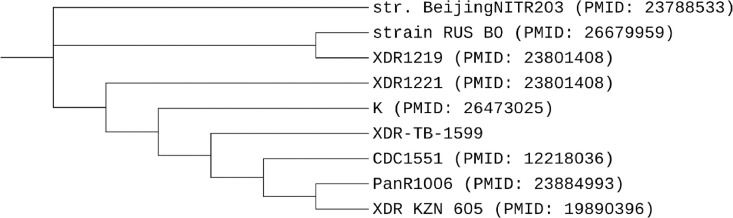
Comparative phylogenetic analysis of *M.tuberculosis* XDR-TB-1599 isolate with different types of *M.tuberculosis* isolates from NCBI database.

Comparative phylogenetic analysis of *M.tuberculosis* XDR-TB-1599 isolate with different types of isolates from NCBI database (Supplementary Table S2) has been performed using CSI Phylogeny tool based on the concatenated alignment of the high-quality SNPs (Fig. 1).

Fig. 1 shows the results of comparative phylogenetic analysis of *M.tuberculosis* XDR-TB-1599 isolate. Completely sequenced genomes of eight MTB strains available through NCBI: str. BeijingNITR203, strain RUS B0, XDR1219, XDR1221, K, CDC1551, PanR1006 and XDR KZN 605 were selected for phylogenetic assessment. Phylogenetic analysis and in silico VNTR revealed (Table 2) that the isolate was distributed into ‘modern’ East Asian lineage (lineage 2).

**Table 2:** Spoligotype, lineage and clade of the XDR *Mycobacterium tuberculosis* clinical isolate.

Isolate	Spoligotype	MIRU-VNTR profile (12 loci)	Lineage	Clade
XDR-TB-1599	000000000003771	223332515353	East Asian	Beijing

The phylogenetic tree was constructed using SNPs data extracted from the genome sequences. The tree is rooted with *M.tuberculosis* H37Rv as outgroup.

Tables 1 and 2, Table S1 show isolate WGS characteristics, spoligotype and lineage definition, and found mutations, respectively.

Table 3 shows the antibiotic resistance of *M.tuberculosis* XDR-TB-1599 isolate.

**Table 3:** Resistance against anti-tuberculosis (TB) drugs according to phenotypic drug susceptibility testing (DST) and whole-genome-sequence-based DST.

Anti-TB drugs	Phenotypic DST	ResFinder	CARD	CASTB	TB-Profiler
Isoniazid	✓	✗	✗	✓	✓
Rifampicin	✓	✓	✗	✓	✓
Ethambutol	✓	✗	✗	✗	✗
Streptomycin	✓	✓	✓	✓	✓
Pyrazinamide	ND	✗	✗	✗	✗
Amikacin	✓	✗	✗	✗	✗
Kanamycin	✓	✗	✗	✗	✗
Ethionamide	✓	✗	✗	✗	✓
Ofloxacin	✓	✗	✗	✗	✗
Capreomycin	ND	✗	✗	✗	✗

ND, not done; NA, not available.

Table S2 shows a list of reference strains with countries where they were received, publications and antimicrobial resistance profile.

MIRU-VNTR and spoligotyping of XDR-TB-1599 *M.tuberculosis* strain identified patterns related to W-Beijing genotype (East Asian lineage) that predominate in Kazakhstan and other Central Asian countries (Table 2) [5]. Many countries around the world noted the spread of W-Beijing family strains of *M.tuberculosis* which was associated with a high risk of drug resistance. The phenotypic drug susceptibility testing showed that XDR-TB-1599 isolate identified as XDR and resistant to first-line (rifampicin, isoniazid, ethambutol, streptomycin) and second-line (ofloxacin, amikacin, capreomycin, ethionamide) anti-TB drugs.

The drug susceptibility testing results were compared with whole-genome sequencing data and annotation results using ResFinder v.3.2 [6]; CARD [7]; CASTB [8], TB-Profiler [9] (Table 3). The whole-genome assembly was screened for mutations associated with resistance to 10 anti-TB drugs, including the first-line drugs isoniazid, rifampicin, ethambutol, pyrazinamide and streptomycin, and the second-line drugs ethionamide, fluoroquinolone, amikacin, capreomycin and kanamycin. Analysis of XDR-TB-1599 *M.tuberculosis* strain for the presence of mutations observed resistance only to rifampicin, isoniazid, streptomycin and ethionamide. Other mutations in genes (Supplementary Table S1) associated with drug resistance to anti-TB drugs were not detected (Ethambutol, amikacin, kanamycin and ofloxacin).

## 2 Experimental Design, Materials and Methods

An extensively drug-resistant clinical isolate of *M.tuberculosis* from new TB case-patient with pulmonary tuberculosis has been collected at the National Research Center for Phthisiopulmonology (Kazakhstan). After primary isolation, *M.tuberculosis* was subcultured on solid Löwenstein-Jensen (LJ) medium. Drug susceptibility testing of *M.tuberculosis* was performed on solid Lowenstein-Jensen medium and using BACTEC-MGIT 960 (BD Diagnostic Systems, USA) system. Spoligotyping was performed on genomic DNA by the gold standard reverse dot-blot spoligotyping method, using a commercially available kit (Ocimum Biosolutions Inc) with positive controls (M.tuberculosis H_37_Ra and *M.bovis*) and negative control and MIRU-VNTR typing using 12 loci (Table 2).

The whole genome of XDR-TB-1599 was sequenced using Roche 454 GS FLX+ Titanium NGS platform. Raw sequencing SFF files were converted to fastq files by SFF converter tool (Galaxy Version 1.0.1). FastQC was applied to analyze reads quality and adapters were trimmed with Trimmomatic v0.38 to truncate low quality reads, filtering for a minimum read length of 36 (parameter: LEADING: 3; TRAILING: 3; SLIDINGWINDOW: 4:20; MINLEN: 36; CROP: 120) and trim low-quality 3′ ends of reads. Nucleotide positions in the reads with a quality score lower than Q20 were removed. Sequencing reads were aligned against the *M. tuberculosis* H37Rv reference genome (GenBank accession: AL123456.3) using BWA 0.6.2. SNPs and InDels were called using GATK tool v.4.1. *De novo* assembly was conducted using Velvet v.1.2.10 and the assembled genome sequence was functionally annotated using the PATRIC platform.

## Ethics Statement

Institutional written informed consent for extraction and collection of sputum for further investigation was signed and obtained from the participated patient. DNA of *M.tuberculosis* XDR-TB-1599 isolate has been extracted from the sputum of the patient. This work was discussed by institutional review board and was approved by the ethical committee of the Center for Life Sciences, National Laboratory Astana, Nazarbayev University (protocol #20, September 22, 2017).

## Credit Author Statement

Asset Daniyarov: Formal analysis, Software, Writing-Original draft preparation.

Askhat Molkenov: Formal analysis, Software, Data curation.

Saule Rakhimova: Investigation, Resources, Funding acquisition.

Ainur Akhmetova: Investigation.

Zhannur Nurkina: Investigation.

Dauren Yerezhepov: Investigation.

Lyailya Chingissova: Resources.

Venera Bismilda: Resources.

Bekzat Toxanbaeva: Resources.

Ainur Akilzhanova: Conceptualization.

Ulan Kozhamkulov: Methodology, Investigation, Project administration, Writing-Original draft preparation, Funding acquisition.

Ulykbek Kairov: Methodology, Formal analysis, Software, Supervision, Writing-Original draft preparation, Funding acquisition.

## Declaration of Competing Interest

The authors declare that they have no known competing financial interests or personal relationships that could have appeared to influence the work reported in this paper.

## Appendix Supplementary materials

Image, application 1
