# Supplementary material for: Whole genome sequence data of Mycobacterium tuberculosis XDR strain, isolated from patient in Kazakhstan
Source: Data Brief. 2020 Oct 17;33:106416. doi: 10.1016/j.dib.2020.106416 (PMC7578676; doi:10.1016/j.dib.2020.106416)
Supplement: Supplementary file 1 [file mmc1.docx]

**Table S1.** Mutations identified by TB-Profiler.

| **Strain** | **INH** | **RIF** | **PZA** | **SM** | **ETH** | **Mutations**  **in candidate genes** |
| --- | --- | --- | --- | --- | --- | --- |
| XDR-TB-1599 | katG Insertion/  deletion found | rpoB Insertion/  deletion found |  | gid Frameshift  (-1 nt) found | ethA Insertion/  deletion found | eis Insertion/deletion found.  eis_promoter Failed to detect the promoter region.  embA Insertion/deletion found.  embA_promoter Failed to detect the promoter region.  embC Insertion/deletion found.  embR Insertion/deletion found.  fabG1 Insertion/deletion found.  gid (c.47T>G)  Substitutions after indel of gid  gid (c.377delA)  gid (c.615A>G)  gyrA Insertion/deletion found.  gyrB Insertion/deletion found.  kasA (c.805G>A, G269S Isoniazid reported to be phenotypically susceptible)  ribD Insertion/deletion found.  rpoC Insertion/deletion found.  rrl Insertion/deletion found.  thyA Insertion/deletion found.  tlyA (c.33A>G) |

INH, isoniazid; RIF, rifampicin; PZA, pyrazinamide; SM, streptomycin; ETH, Ethionamide.

**Table S2.** List of reference strains with countries where they were received, publications and antimicrobial resistance profile.

| Isolate | Country | PMID | Antibiotic resistance profile |
| --- | --- | --- | --- |
| str. Beijing/NITR203 | India | 23788533 | - |
| RUS_B0 | Russian Federation | 26679959 | - |
| XDR1219 | China | 23801408 | XDR |
| XDR1221 | China | 23801408 | XDR |
| K | South Korea | 26473025 | susceptible |
| CDC1551 | USA | 12218036 | - |
| PanR1006 | South Africa | 23884993 | - |
| XDR KZN 605 | South Africa | 19890396 | XDR |
| H37Rv | UK | 9634230 | - |
